# Motility-dependent processes in *Toxoplasma gondii* tachyzoites and bradyzoites: same same but different

**DOI:** 10.1128/msphere.00855-24

**Published:** 2025-02-12

**Authors:** Robyn S. Kent, Gary E. Ward

**Affiliations:** 1 Department of Microbiology and Molecular Genetics, University of Vermont Larner College of Medicine, Burlington, Vermont, USA; 2 Department of Microbiology and Immunology, University of Oklahoma Health Sciences Center, Oklahoma City, Oklahoma, USA; Institut Pasteur de Montevideo, Montevideo, Uruguay

**Keywords:** *Toxoplasma gondii*, bradyzoite, motility, apicomplexan parasites, tachyzoite, chronic infection

## Abstract

During infection, *Toxoplasma gondii* tachyzoites must be able to move in order to migrate through tissues, cross biological barriers, and penetrate into and egress from cells they infect. Bradyzoite-stage parasites, which establish infection in naïve hosts, also require motility to escape from cysts after they are ingested and to subsequently migrate to the gut wall, where they either invade cells of the intestinal epithelium or squeeze between these cells to infect the underlying tissue. Little is known about the motility of bradyzoites, which we analyze in detail here and compare to the well-characterized motility and motility-dependent processes of tachyzoites. Unexpectedly, bradyzoites were found to be as motile as tachyzoites in a three-dimensional model extracellular matrix, and they showed increased invasion into and transmigration across monolayers of certain cell types, consistent with their need to establish infection in the gut. The motility of the two stages was inhibited to the same extent by cytochalasin D and KNX-002, compounds known to target the parasite’s actomyosin-based motor. Other compounds that impact tachyzoite motility (tachyplegin and enhancer 5) have a reduced effect on bradyzoites. Furthermore, rapid bradyzoite egress from infected cells is not triggered by treatment with calcium ionophores, as it is with tachyzoites. The similarities and differences between these two life cycle stages highlight the need to characterize both tachyzoites and bradyzoites for a more complete understanding of the role of motility in the parasite life cycle and the effect that motility-targeting therapeutics will have on disease establishment and progression.

**IMPORTANCE:**

*Toxoplasma gondii* is a parasite that chronically infects around one-third of the world's population. *Toxoplasma* uses motility for multiple purposes during infection, including extracellular migration, invasion into host cells, and host cell egress. These motility-dependent processes have been extensively studied in the life cycle stage responsible for acute infection, the tachyzoite. In contrast, motility and motility-dependent processes are poorly understood in bradyzoite-stage parasites, which are responsible for both establishing infection after consumption of infected meat and initiating potentially life-threatening reactivated infections in the brains of immunocompromised individuals. We show here that the motility and motility-dependent processes of bradyzoites are similar in many respects to those of tachyzoites but markedly different in others. The results of this study highlight the need to consider both life cycle stages in attempts to develop drugs targeting parasite motility and the signaling processes that regulate motility-dependent processes during infection by these important human pathogens.

## INTRODUCTION

*Toxoplasma gondii* is a prevalent human pathogen, infecting >30% of the global human population (1–4). Infection with *T. gondii* mostly occurs via ingestion of either bradyzoite-containing tissue cysts in undercooked meat or sporozoite-containing oocysts in cat feces that can also contaminate water sources (5). While acute infection is usually subclinical in immunocompetent individuals, it can cause ocular (6, 7) and pulmonary (8) disease particularly associated with virulent atypical strains (9).

Bradyzoites, after excysting from tissue cysts, must efficiently migrate to the gut wall and either actively invade the gut epithelium or penetrate between these cells to access the lamina propria (10). Following infection, the bradyzoites transition into the fast-replicating tachyzoite stage. The tachyzoites disseminate throughout the body and, following repeated rounds of invasion, replication, and egress from host cells, they elicit and are largely cleared by the host immune response (11). However, a subset of the tachyzoites differentiate into bradyzoites and build a protective cyst wall, primarily in the brain and skeletal muscle, to establish a chronic infection (12). Bradyzoites within these intracellular tissue cysts replicate much more slowly than tachyzoites (13, 14) and have traditionally been considered more dormant, although recent data show that the cysts grow and the parasites within replicate *in vivo* (15). The cysts are resistant to all current therapeutics (16) and can establish an infection in a new host if ingested (12). Cysts can also reactivate *in situ* if the host becomes immunocompromised (17, 18), causing potentially life-threatening disease.

Much is known about motility of the tachyzoite stage (19–21), including the composition of the motor complex thought to power motility and the role of several proteins that link the parasite motor to the substrate, along which the parasite moves (22–26). Furthermore, we know that tachyzoite motility is important not only for parasite movement between cells and through tissues but also for other essential aspects of the lytic cycle, such as invasion into and egress from host cells (25, 27). In contrast, very little is known about motility and motility-dependent processes of the bradyzoite stage, although motility is clearly critical for establishing infection after the ingestion of tissue cysts. Tachyzoites are often referred to as the “highly motile” stage, a viewpoint consistent with the recent report that type II strain ME49 bradyzoites chemically liberated from cysts show reduced motility in two-dimensional (2D) assays compared to mechanically liberated tachyzoites (28). A more systematic comparison of the motile behavior and motility mechanisms of these two life cycle stages will shed new light on the biological processes underlying *Toxoplasma* infection.

We show here that, after excystation using a brief protease treatment and mechanical shear, bradyzoites of another type II strain, prugnaud (PRU), are in fact as motile as tachyzoites within a three-dimensional (3D) model extracellular matrix, show enhanced invasion into two gut cell lines, and surpass tachyzoites in their ability to transmigrate across certain polarized epithelial cell monolayers. Bradyzoites and tachyzoites respond similarly to treatment with some known modulators of tachyzoite motility, and differently to others. Finally, we confirm previous work showing that—in contrast to tachyzoites—calcium stimulation does not lead to simultaneous, rapid egress of bradyzoites from infected host cells. Together, these data demonstrate that while there are similarities in the motility of tachyzoites and bradyzoites, there are also stage-specific differences that will need to be considered in current efforts to therapeutically target motility-dependent processes (29–33) in these widespread human and animal pathogens.

## MATERIALS AND METHODS

### Cell and parasite culture

Tachyzoite stage parasites (RH, PRU-RFP, and PRU-LDH2GFP) were propagated by serial passage in a primary fibroblast-like cell line from human foreskin provided by Dr. Thomas Moehring. These human foreskin fibroblasts (HFFs) were grown to confluence in Dulbecco’s modified Eagle’s medium (DMEM) (Life Technologies, Carlsbad, CA) containing 10%(vol/vol) heat-inactivated fetal bovine serum (FBS) (Life Technologies), 10 mM HEPES, pH 7, and 100 units/mL penicillin and 100 µg/mL streptomycin, as previously described (34). Prior to infection with *T. gondii*, the medium was changed to DMEM supplemented with 10 mM HEPES, pH 7, 100 units/mL penicillin and 100 µg/mL streptomycin, and 1% (vol/vol) FBS (hereafter referred to as 1% DMEM). Differentiation of the parasites to the bradyzoite stage was induced by replacing the medium with differentiation medium (RPMI supplemented with 10 mM HEPES, pH 8.2, 100 units/mL penicillin and 100 µg/mL streptomycin, 2 mM L-glutamine and 1% [vol/vol] FBS) 1–2 h after challenging with tachyzoites and growth in ambient CO_2_ conditions, as previously described (35). For all *in vitro* bradyzoite assays, the differentiation medium was replaced daily for a minimum of 5 days before parasite isolation. The cell lines used for invasion and transmigration assays were authenticated as follows: authenticated Hs27 HFFs, HCT-8 cells, and EA.Hy926 cells were purchased directly from American Type Culture Collection. Madin-Derby canine kidney (MDCK) cells were authenticated by short tandem repeat (STR) profiling by IDEXX (Westbrook, ME), and CACO-2 cells were authenticated by STR profiling at the Vermont Integrative Genomics Resource (Burlington, VT).

### Mouse infections

Infections were carried out under University of Vermont Institutional Animal Care and Use Committee protocol PROTO202000126 using 4- to 5-week-old male CBA/J mice purchased from Jackson Laboratories (Bar Harbor, ME). All mice were socially housed and acclimated for at least 3 days prior to infections. Mice (two mice per biological replicate were pooled per motility experiment) were challenged by intraperitoneal injection of 100 PRU-LDH2GFP parasites resuspended in a total volume of 200 µL sterile phosphate-buffered saline (PBS). Daily animal health monitoring began 2 days after infection. Mice were euthanized if pre-determined welfare thresholds were exceeded.

### Isolation of bradyzoites from infected mice

Parasites were harvested for assays 28–32 days after infection. Brains were washed in 5 mL PBS before homogenization in PBS (2 mL/brain) using a Dounce homogenizer. Two brains per sample were pooled before serially passing through 18G, 20G and 22G needles five times and were centrifuged at 2,000 *× g* for 10 min. Each sample was thoroughly resuspended in 6 mL 20% dextran-150 in PBS and centrifuged at 400 *× g* for 10 min. The myelin layer and dextran-150 were removed by aspiration. The pellet was washed once with 10 mL PBS by centrifugation at 2,000 × *g* for 10 min. To excyst the bradyzoites, the pellet was resuspended in 1 mL acid pepsin (AP; stock solution [0.01 mg/mL pepsin in 1% NaCl, pH 2.1] diluted in 1 mL freshly made 1% NaCl immediately before use) and incubated at 37°C for 1 min. The suspension was then passed through a 26G needle to mechanically disrupt the cyst wall, directly into 2 mL Na_2_CO_3_ and 2 mL 1% DMEM to inactivate the pepsin. The released parasites were filtered through a 3 µm Whatman Nuclepore filter (Millipore Sigma), pelleted (5 min at 2,000 × *g*), and resuspended in motility buffer (21) (1× minimum essential medium lacking sodium bicarbonate, 1% [vol/vol] FBS, 10 mM HEPES, pH 7.0, and 10 mM GlutaMAX L-alanyl-L-glutamine dipeptide) for motility assays.

### Isolation of bradyzoites from cell culture

Bradyzoites were isolated by passing infected cells through a 22G needle, centrifugation (2,000 × *g,* 5 min), resuspension in 1 mL AP, incubation at 37°C for 1 min, mechanical disruption of the cyst wall using a 26G needle, inactivation of the pepsin in 2 mL Na_2_CO_3_ and 2 mL 1% DMEM, as above. The parasites were then filtered through a 3 µm Whatman Nuclepore filter (Millipore Sigma), pelleted (5 min at 2,000 *× g*), and resuspended in motility buffer for motility assays or 1% DMEM for invasion and transmigration assays, as outlined below.

### Isolation of tachyzoites from cell culture

Tachyzoites were isolated from infected HFF cells by syringe release through a 26G needle, filtering through a 3 µm Whatman Nucleopore filter (Millipore Sigma), centrifugation (2,000 × *g,* 5 min), and resuspension in motility buffer (21) (for motility assays) or 1% DMEM (for invasion and transmigration assays) as outlined below.

### Parasite motility assays

3D motility assays were performed as previously described (21). Briefly, isolated parasites were resuspended in motility buffer (21) and maintained on ice. For each motility capture, parasites (2.5 µL) were combined with motility buffer (7.5 µL) with or without drug and Matrigel (7.5 µL) and gently mixed. This mixture was transferred to a Pita chamber before incubating at 27°C for 7 min. The final drug concentration within the 15 µL assay volume is the concentration reported here. The Pita chamber was then transferred to the heated microscope stage for 3 min at 35°C. Images of 1,024 × 384 pixels were captured over 80 s using an iXON Life 888 EMCCD camera (Andor Technology) driven by NIS Elements software v.5.11 (Nikon Instruments). Image stacks consisted of 41 z-slices, captured 1 μm apart with a 16 ms exposure time. Tachyzoites were tracked using Hoechst 33242 fluorescence, and bradyzoites were tracked using cytosolic green fluorescent protein (GFP) fluorescence. Tracking was achieved with Imaris ×64 v.9.2.0 (Bitplane AG, Zurich, Switzerland). The minimum displacement cutoff thresholds were determined using heat-killed parasites (as previously described [21]) to be 2.5 and 2.8 µm for tachyzoites and bradyzoites, respectively.

### Invasion assays

The invasion of tachyzoites (PRU-RFP) and bradyzoites (PRU-LDH2GFP) was measured in co-invasion assays, where both stages were applied to the same monolayer to reduce data variability. Bradyzoites and tachyzoites were independently isolated (as above) and combined at a ratio of approximately 1:1. Each biological replicate challenged a panel of host cells with the same mixture of tachyzoites and bradyzoites; Hs27 HFF cells were always included as a control. Cells were grown on 18 mm coverslips pre-coated with poly-l-lysine (0.01%) until confluent; added parasites were therefore presented with target cells occupying equal surface areas, regardless of the size or shape of the cell types utilized. For MDCK, EA.HY926, HCT-8, and CACO-2 cells, confluency and formation of tight junctions were confirmed by immunofluorescence assays against ZO-1 (see below).

Each coverslip containing a confluent host cell monolayer was challenged in the well of a 12-well plate with 1 mL parasites resuspended in 1% DMEM (0.4–1.1 × 10^5^ total parasites, for a multiplicity of infection of one to three parasites per cell) for 1 h at 37°C in 5% CO_2_. The exact starting number of bradyzoites and tachyzoites in the challenge suspension was quantified by counting RFP-expressing (tachyzoites) and GFP-expressing (bradyzoites) parasites. After the 1 h incubation, the monolayers were gently washed with 1 mL PBS, fixed with 2% PFA for 15 min, and blocked for 30 min with PBS containing 1% (wt/vol) bovine serum albumin (PBS-BSA). Extracellular parasites were stained by incubation for 60 min with anti-SAG3 mouse monoclonal antibody (#NR-50257, BEI Resources) diluted 1:500 in PBS-BSA. After washing three times with PBS-BSA, coverslips were incubated with either Alexafluor 405 nm- or Alexafluor 647 nm-conjugated anti-mouse secondary antibody (Invitrogen), diluted 1:500 in PBS-BSA, in the dark for 1 h. To quantify the number of invaded parasites, 10 fields of view were imaged per biological replicate. Intracellular parasites were identified as Alexafluor 405/647 negative and positive for either RFP fluorescence (540 nm excitation/605 nm emission, tachyzoites) or GFP fluorescence (488 nm excitation/534 nm emission, bradyzoites). Within each biological replicate, the relative number of invaded tachyzoites and bradyzoites was normalized using the measured ratio of tachyzoites and bradyzoites in the starting suspension. For example, if 0.5 × 10^5^ bradyzoites were added together with 0.6 × 10^5^ tachyzoites, the number of invaded bradyzoites would be multiplied by 1.2. To normalize between biological replicates, the total number of parasites added to the monolayer was normalized to the greatest total number of parasites after within-replicate normalization. For example, if replicate 1 used a total of 1 × 10^5^ parasites in the starting suspension while replicate 2 used 2 × 10^5^, the number of invaded parasites in replicate 1 would be multiplied by 2. Finally, the normalized invasion numbers for each cell type are presented relative to the invasion into HFF cells.

### Transmigration assays

MDCK, HCT-8, or CACO-2 cells were grown to confluence on 3 µm ThinCert Cell Culture Inserts (Griener Bio) in 12-well plates. Confluence and monolayer polarization were confirmed prior to experiments by measuring transcellular electrical resistance (TCER) and performing immunofluorescence assays on the tight junction protein ZO-1 (data not shown). Barrier integrity is reached after TCER plateaus and stabilizes at >2,000 Ω/cm^2^ and >125 Ω/cm^2^ for MDCK/HCT- 8 and CACO-2 cells, respectively (36–38). Stable TCER was confirmed for 2 days prior to challenge with parasites. The number of days required to achieve stable TCER without overgrown monolayers was different for the different cell types tested; staggered cell seeding was therefore necessary. In each biological replicate, cells were challenged with either no parasites (unchallenged), tachyzoites (0.5–1.0 × 10^6^), or bradyzoites (0.1–0.5 × 10^6^) for 6 h. TCER measurements were made for each monolayer immediately after the addition of parasites and 6 h later. In a separate experiment where parasite transmigration was not quantified, tachyzoites (0.5–1.0 × 10^6^) or bradyzoites (0.1–0.5 × 10^6^) and fluorescein isothiocyanate (FITC)-dextran (MW 3,000 Da, 1 mg/mL; Molecular Probes) in PBS were added to cell monolayers grown on ThinCert Cell Culture Inserts. The passage of FITC-dextran across the monolayer was quantified after 6 h using a Biotek Synergy 2 plate reader. The relative fluorescence intensity (RFI) of the dextran that had crossed the cell barrier was divided by the RFI of the dextran added to the top of the monolayer and multiplied by 100 to quantify the percentage of the added dextran that had crossed at some time within the 6 h challenge. One monolayer for each cell type per experiment was treated with 2 µg/mL CytoD to disrupt the tight junctions, resulting in reduced TCER and rapid flux of dextran and parasites across the cells and into the lower chamber of the well (39).

To quantify transmigration across the monolayers, the number of parasites per microliter in the challenge suspension and in the lower chamber of the well 6 h after challenge was quantified using a Miltenyl MACSQuant VYB. Bradyzoites and tachyzoites were distinguished based on RFP (PRU-RFP tachyzoites) or GFP (PRU-LDH2 GFP bradyzoites) fluorescence, respectively. The proportion of bradyzoites and tachyzoites able to transmigrate was normalized based on the numbers of parasites in the challenge suspension and expressed as a percentage of those added to the upper chamber.

### Egress assays

Infected cells containing mature tachyzoite vacuoles (~48 h post-inoculation) or bradyzoite cysts (5–6 days post-differentiation) were incubated with 1 µM ionomycin or 1 µM A23187 to stimulate egress. For tachyzoites, monolayers were fixed 2.5, 5.0, and 30.0 min after stimulation by gently replacing the media with 2% PFA in PBS for 10 min. For bradyzoites, monolayers were fixed 5, 30, 60, and 120 min following stimulation by gently replacing the media with 2% PFA in PBS for 10 min. For each biological replicate, 100 vacuoles were scored as either intact or egressed based on the dispersal of tachyzoites (PRU-RFP) or bradyzoites (PRU-LDH2 GFP).

## RESULTS

### Type II (PRU) tachyzoites are as motile as type I (RH) tachyzoites

3D motility has only been characterized in type I (RH strain) tachyzoites and mutant lines made in this background (21, 40–43). However, type II strains (e.g., PRU) are much more efficient than type I at generating bradyzoites, both *in vitro* and in mice, and therefore were preferred for a comparison of bradyzoite and tachyzoite motility. We first assessed how well PRU tachyzoites move in our established 3D motility assay (21). In contrast to what was previously observed in 2D motility assays with type II Me49 tachyzoites (44), type II PRU tachyzoites were as motile in the 3D motility assay as type I RH tachyzoites, which could be due to either assay- or strain-specific differences. We saw no differences in the proportion of PRU versus RH tachyzoites moving in 3D, the distance they moved (both first-to-last point displacement and track length), or their maximum and average speeds along the trajectory (Fig. S1). Thus, type II PRU strain parasites can serve as a good model to directly compare bradyzoite and tachyzoite motilities using the 3D motility assay.

### Bradyzoites are as motile as tachyzoites in a 3D model extracellular matrix

As bradyzoites and tachyzoites are morphologically indistinguishable in the 3D motility assay, we utilized the PRU-derived fluorescent parasite reporter line, LDH2::GFP, in which GFP placed under the control of the *ldh2* stage-specific promoter is expressed exclusively in bradyzoites (45), to specifically identify and track bradyzoites (21) by their GFP expression. Motility of the non-GFP-expressing tachyzoites is tracked by staining the nucleus with Hoechst 33342 (as in Fig. S1 and reference 21).

**Fig 1:**
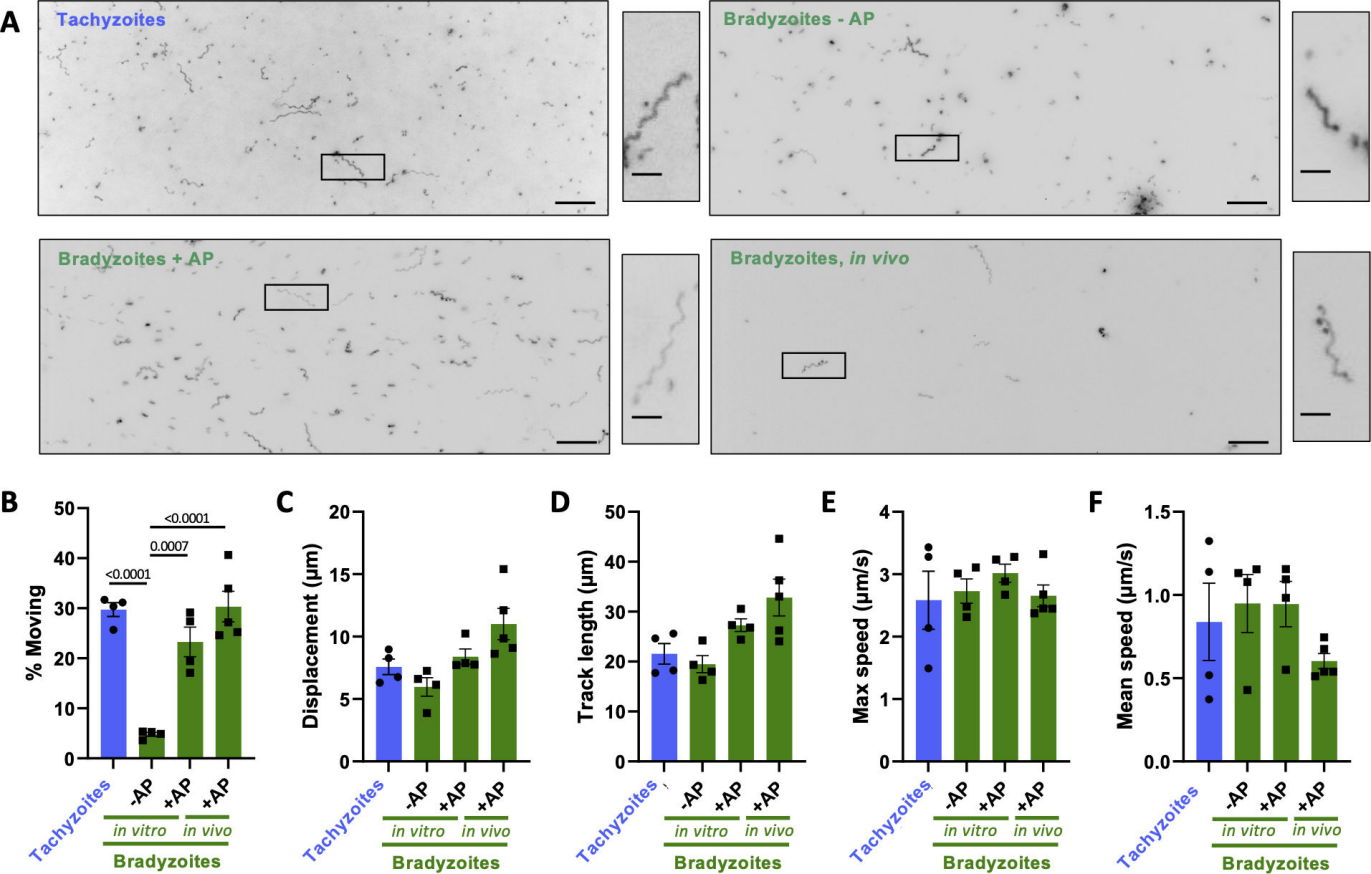
Comparison of the motility of PRU tachyzoites and bradyzoites. (**A**) Representative maximum intensity projections of parasites moving through Matrigel during 60 s of imaging; scale bar = 40 µm. Insets (black boxes) are magnified, rotated, and displayed to the right of each full field of view; scale bar = 10 µm. (**B**) Percentage of tachyzoites moving with displacements of >2.5 µm and bradyzoites moving with displacements of >2.8 µm during 60 s of imaging. (**C–F**) For all parasites that exceeded the 2.5/2.8 µm displacement thresholds (see Materials and Methods), the following median trajectory parameters were quantified: (C) displacement, (**D**) track length, (**E**) maximum speed achieved across the entire track, and (F) mean speed. On the graphs, each data point represents a biological replicate consisting of three to six technical replicates. Bar height shows the mean, and error bars show the s.e.m. of the biological replicates. The numbers of parasites analyzed in panels B–F were 4,387 (tachyzoites), 1,132 (bradyzoites – AP), 3,757 (bradyzoites + AP), and 728 (bradyzoites, *in vivo*). Stage- and isolation method-specific differences in the motility parameters were compared using an ordinary one-way analysis of variance with Tukey’s correction for multiple comparisons; only statistically significant differences (*P* < 0.05) are shown.

First, we evaluated the motility of bradyzoites isolated from *in vitro* cultures where tachyzoite-to- bradyzoite differentiation was induced by standard stress conditions (pH 8.2, ambient CO_2_) (35). We compared the well-characterized 3D motility of *in vitro-*derived tachyzoites to that of bradyzoites exposed to stress for 5 days. The bradyzoites were liberated from their surrounding cyst wall and host cell by mechanical disruption either with or without a 1 min pre-digestion with acid pepsin (+AP and −AP, respectively; Fig. 1). Using the 3D motility assay to analyze hundreds of parasites, we found no significant difference in the ability of tachyzoites and bradyzoites isolated with AP to move (compare the two left panels in Fig. 1A and the first and third bars in Fig. 1B). Without AP digestion, we recovered only 30% the number of bradyzoites recovered with AP digestion, presumably due to retention within the cyst, and significantly fewer of the −AP bradyzoites moved (compare second and third bars in Fig. 1B). We then compared the trajectory characteristics of those tachyzoites and bradyzoites (+AP and −AP) that moved. There were no significant differences between the tachyzoite and either of the bradyzoite preparations in parasite displacement (Fig. 1C, first three bars), track length (Fig. 1D), and maximum (Fig. 1E) and mean (Fig. 1F) speeds achieved. Bradyzoites can therefore move as effectively as tachyzoites in 3D, and a brief digestion of the cyst wall by acid pepsin yields more motile bradyzoites from *in vitro* culture than mechanical disruption alone.

To ascertain if the motility of our *in vitro-*derived bradyzoites is representative of mature bradyzoites found in cysts *in vivo*, we also characterized the motility of bradyzoites isolated from the brains of chronically infected mice. The 3D motility assay requires parasite preparations relatively free from cellular contamination. Isolation of free bradyzoites from mouse brain to a sufficient level of purity is inefficient and yields are low: the number of bradyzoites isolated from the brains of two mice is equivalent to ~4% of the number that can be obtained from one T75 culture flask of *in vitro-*differentiated parasites. Nevertheless, we recovered sufficient numbers of bradyzoites from the mouse brain to compare to *in vitro-*differentiated bradyzoites liberated from cysts (+AP). We saw no significant difference in the proportion of parasites that move (Fig. 1A, bottom two panels; Fig. 1B, right two bars). Comparing how far and fast these two bradyzoite populations move also reveals no significant differences in their median displacement, track length, or maximum and mean speeds (Fig. 1C through F).

In addition to quantifying and comparing the average motility parameters, we analyzed the distributions of each of the motility parameter measurements in the different parasite preparations to determine if the behavior of parasites across the population was consistent. We compared the 5%–95% range that describes the motility of 90% of all parasites characterized and found no significant differences within this range for displacement, track length, or maximum and mean speeds (Fig. S2A through D).

These data demonstrate that, using the isolation method reported here, bradyzoites are motile and their motility is equivalent, in all aspects measured, to tachyzoites. Furthermore, 5 day stress-induced bradyzoites from *in vitro* culture (isolated +AP) faithfully recapitulate the motility of bradyzoites isolated from the brains of infected mice, providing a more abundant source of parasites for studies of bradyzoite motility without having to use an animal model.

### Bradyzoites invade intestinal cells to a greater extent than tachyzoites

Because parasite motility plays a critical role in host cell invasion, we next determined if excysted bradyzoites were able to invade host cells as efficiently as tachyzoites and if either stage showed host cell specificity. To this end, we developed a co-invasion assay in which approximately equal numbers of PRU-RFP tachyzoites (46) and PRU-LDH2::GFP bradyzoites (45) are mixed and used to challenge the same monolayer. Extracellular parasites are identified and excluded from quantification by staining the non-permeabilized samples for surface protein SAG3, which is expressed by both tachyzoites and bradyzoites (47). The cell types tested were HFFs; EA.hy926 (human vascular endothelial) cells; MDCK cells; and two intestinal epithelial cell lines, CACO-2 and HCT-8.

**Fig 2:**
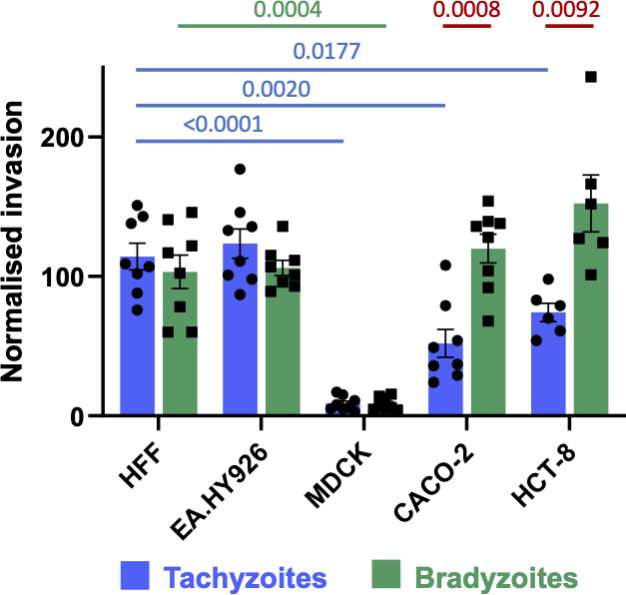
Impact of parasite stage and host cell type on invasion efficiency. Tachyzoite (blue) and bradyzoite (green) invasion into monolayers of different cell types was determined by co-invasion assay. The data were normalized to the number of each parasite stage added to the well and the mean invasion by that stage (see Materials and Methods) into HFF cells. Invasion efficiency of tachyzoites (blue bars) and bradyzoites (green bars) into each monolayer type was compared to invasion into confluent HFFs using an ordinary one-way analysis of variance with Tukey’s correction for multiple comparisons; significant differences are noted above the graph (blue lines, tachyzoites; green lines, bradyzoites). Invasion by the different stages into the same monolayer type was compared by unpaired Student’s *t*-tests; significant differences are noted above the graph (red lines).

Compared to their levels of invasion into HFF and EA.hy926 cell monolayers, tachyzoites showed reduced invasion into CACO-2 and HCT-8 monolayers (50% and 60%, respectively) and much reduced invasion into MDCK monolayers (~7.5%) in 60 min invasion assays (Fig. 2). With bradyzoites, invasion into MDCK cell monolayers is also reduced (~8%) compared to HFF monolayers, but, in contrast to tachyzoites, bradyzoites invade CACO-2 and HCT-8 cell monolayers at levels comparable to HFF monolayers (Fig. 2). Thus, bradyzoites invade intestinal epithelial cell monolayers more than twice as efficiently as tachyzoites, while no significant differences in invasion were observed for the other cell types tested.

### Bradyzoites transmigrate across MDCK but not CACO-2 polarized monolayers more efficiently than tachyzoites

**Fig 3:**
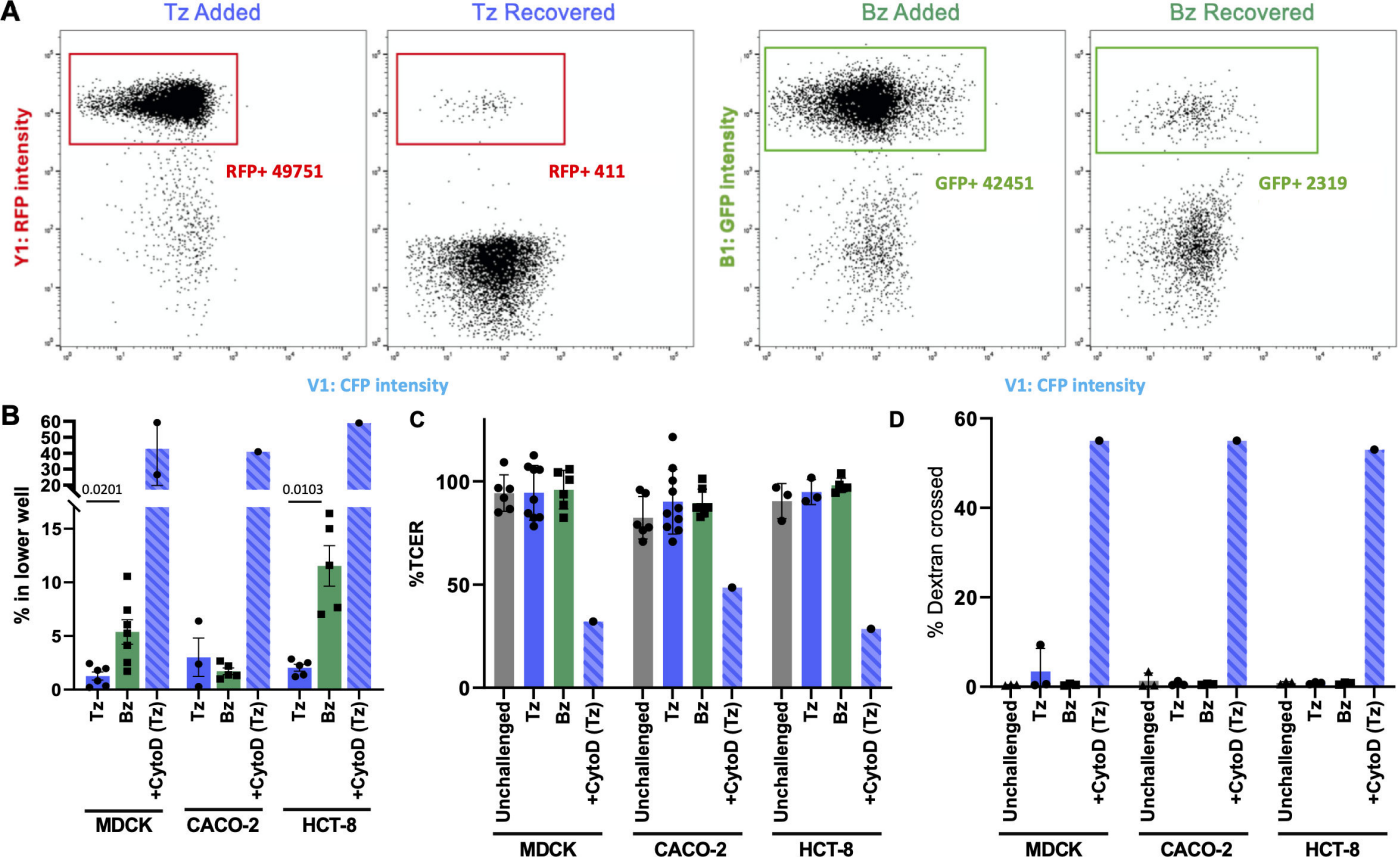
Bradyzoites transmigrate more efficiently than tachyzoites across MDCK and HCT-8 polarized monolayers, without altering barrier integrity. (**A**) Representative flow panels showing the gating strategy used to identify tachyzoites (left two panels, Tz, RFP+) and bradyzoites (right, Bz, GFP+). The strategy is used to quantify the number of parasites added to (added) and transmigrated across (recovered) a CACO-2 cell monolayer in 6 h. (**B**) Quantification of the number of parasites that had crossed MDCK (left), CACO-2 (middle), or HCT-8 (right) monolayers and were recovered in the bottom well of the chamber within 6 h of challenge, expressed as the percentage of parasites initially added to the upper chamber. Addition of 2 µg/mL cytochalasin D prior to tachyzoite challenge (CytoD Tz) disrupted the tight junctions of the monolayers and allowed 40%–60% of the parasites to fall through the cell culture insert and into the lower chamber. Tachyzoite versus bradyzoite transmigration was compared for each cell line using Student’s *t*-test; only significant differences are indicated. (**C**) The transcellular electrical resistance (TCER) prior to and 6 h after challenge revealed no change due to parasite addition, unless the monolayers were intentionally disrupted with CytoD. The data were normalized to the average TCER measurement for all unchallenged replicates. TCER values were compared using an ordinary one-way analysis of variance (ANOVA) and Tukey’s correction for multiple comparisons. No significant differences were detected except in the samples that were treated with CytoD. (D) Cumulative dextran flux across the monolayers during the entire 6 h challenge was quantified to determine if any transient changes in barrier integrity occurred. Dextran was only detected in the lower chamber when the monolayers were intentionally disrupted with CytoD. The data were analyzed using an ordinary one-way analysis of variance and Tukey’s correction for multiple comparisons.

Establishing infection in the gut involves either invasion of and replication within intestinal epithelial cells or transmigration across the gut epithelium to access the lamina propria directly (10, 44, 48). Having shown that tachyzoites and bradyzoites invade intestinal epithelial cells with differing efficiencies, we then tested whether there are differences in their ability to transmigrate across epithelial cell monolayers. First, we developed a transmigration assay that directly quantifies transmigration across the monolayer without coupling transmigration to subsequent host cell invasion as in previously described assays (44, 48). We established a robust gating strategy to distinguish PRU-RFP tachyzoites and PRU-LDH2::GFP bradyzoites by flow cytometry (Fig. 3A) and used this strategy to quantify the number of tachyzoites and bradyzoites that were able to transmigrate across an intact polarized monolayer of MDCK, HCT-8, or CACO-2 cells grown on ThinCert cell culture inserts. The number of parasites recovered in the bottom well of the plate was then normalized to the number of parasites added to the monolayer, as quantified by flow cytometry, to compare transmigration efficiency.

Bradyzoites exhibited significantly greater transmigration across MDCK and HCT-8 monolayers than tachyzoites (4.0- and 5.5-fold increases in transmigration efficiency, respectively; Fig. 3B). In contrast, we saw no significant difference between the number of bradyzoites and tachyzoites crossing a CACO-2 monolayer (Fig. 3B). To determine if either parasite stage compromised the barrier integrity of the monolayers, we monitored transcellular electrical resistance (TCER) and saw no measurable decrease in resistance after 6 h of challenge (Fig. 3C), indicating no loss of barrier integrity when challenged with either bradyzoites or tachyzoites. As a positive control for barrier disruption, we pre-treated monolayers with 2 µg/mL cytochalasin D (CytoD) to disrupt tight junction integrity (39), prior to addition of the parasites. CytoD treatment also inhibits the motility of both tachyzoites (49, 50) and bradyzoites (see below). As expected, CytoD treatment led to rapid depolarization of the monolayer (Fig. 3C) and, once the monolayer was disrupted, more than half of the non-motile parasites fell through the cell culture insert into the bottom well of the chamber (Fig. 3B). We further confirmed that the barriers had not become transiently leaky during the 6 h assay window by monitoring dextran flux across the monolayers. We saw no passage of fluorescent dextran from the top to the bottom well throughout the course of the experiment, while in monolayers treated with CytoD dextran fluorescence reached equilibrium between the two sides of the cell monolayer within 6 h (Fig. 3D). These data confirm and build upon previous reports demonstrating the ability of tachyzoites to transmigrate across MDCK (44), CACO-2 (48) and m-ICcl2 (48) cell monolayers, and show that enhanced bradyzoite transmigration across MDCK and HCT-8 monolayers reported here does not result from a change in monolayer integrity.

### Pharmacological modulators of tachyzoite motility have both similar and distinctly different effects on bradyzoite motility

#### CytoD and KNX-002

**Fig 4:**
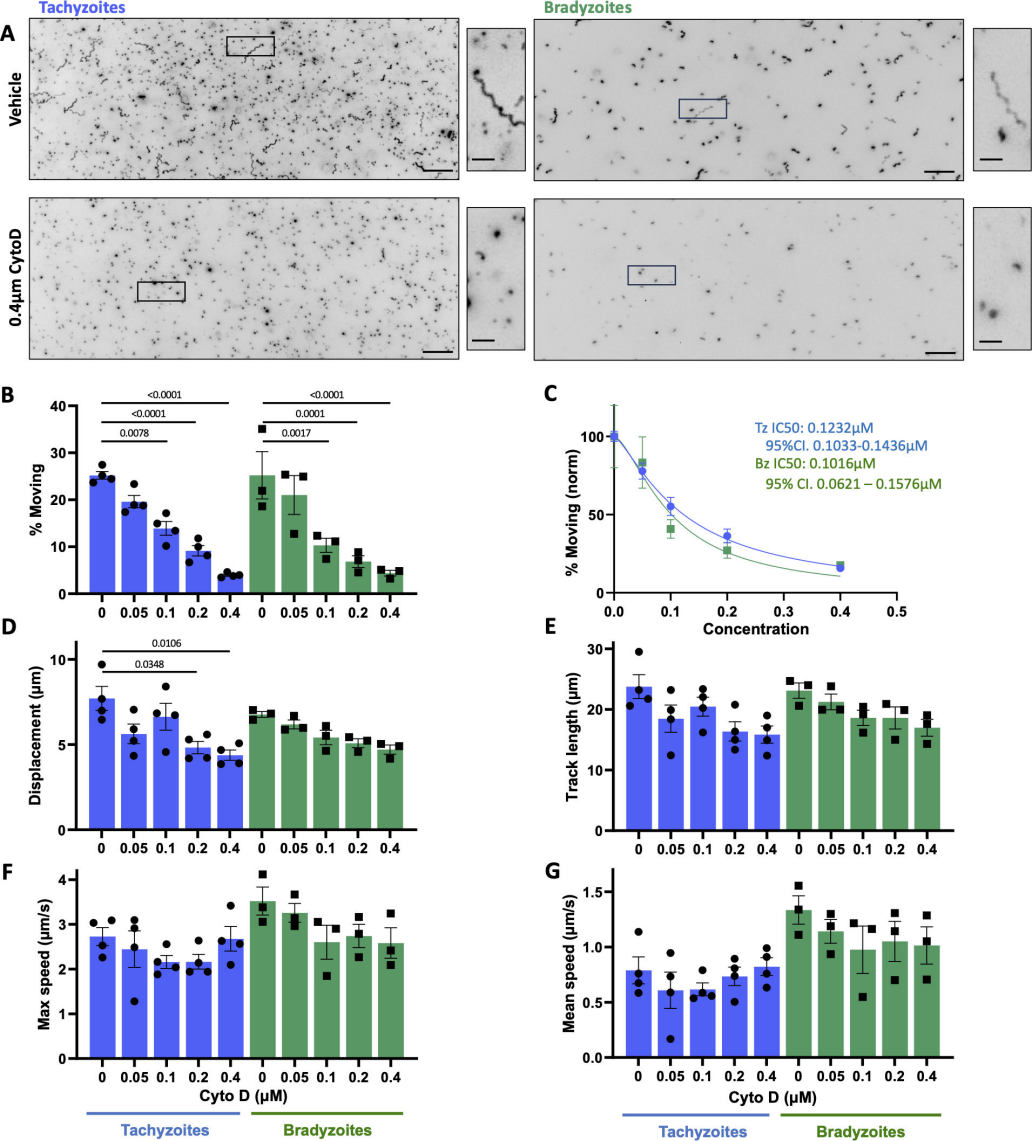
Comparison of tachyzoite and bradyzoite motility in the presence of cytochalasin D (CytoD). (**A**) Representative maximum intensity projections of tachyzoites and bradyzoites moving through Matrigel during 60 s of imaging in the absence (top two panels) or presence (bottom two panels) of 0.4 µM CytoD; scale bar = 40 µm. Insets (black boxes) are magnified, rotated, and displayed to the right of each full field of view; scale bar = 10 µm. (**B**) Percentage of tachyzoites moving >2.5 µm and bradyzoites moving >2.8 µm during 60 s of imaging. (**C**) The IC50 values for the data shown in panel B were calculated for tachyzoites (blue) and bradyzoites (green); % motility for each data set was normalized to 100% in the sample incubated without CytoD. No significant difference was seen in the tachyzoite versus bradyzoite response to treatment, as indicated by the overlapping 95% confidence intervals (CIs) of the calculated IC50 values. (**D–G**) For all parasites that exceeded the 2.5/2.8 µm displacement thresholds, the following median trajectory parameters were quantified: (D) displacement, (**E**) track length, (**F**) maximum speed, and (G) mean speed. On the graphs, each data point represents a biological replicate consisting of two to three technical replicates. Bar height shows the mean, and error bars show the s.e.m. of the biological replicates. The responses of tachyzoites and bradyzoites at each concentration of CytoD were compared using unpaired Student’s *t*-tests; no significant differences were identified between the two stages at any dose. The numbers of parasites analyzed in panels B–G were 4,129; 989; 2,440; 1,086; and 906 (tachyzoites 0, 0.05, 0.1, 0.2, and 0.4 µM CytoD, respectively); and 2,529; 1,365; 1,786; 1,109; and 568 (bradyzoites 0, 0.05, 0.1, 0.2, and 0.4 µM CytoD, respectively). The responses of tachyzoites or bradyzoites to treatment with each concentration of compound were compared to the vehicle control (0) using an ordinary one-way analysis of variance and Tukey’s correction for multiple comparisons; only statistically significant differences (*P* < 0.05) are shown.

**Fig 5:**
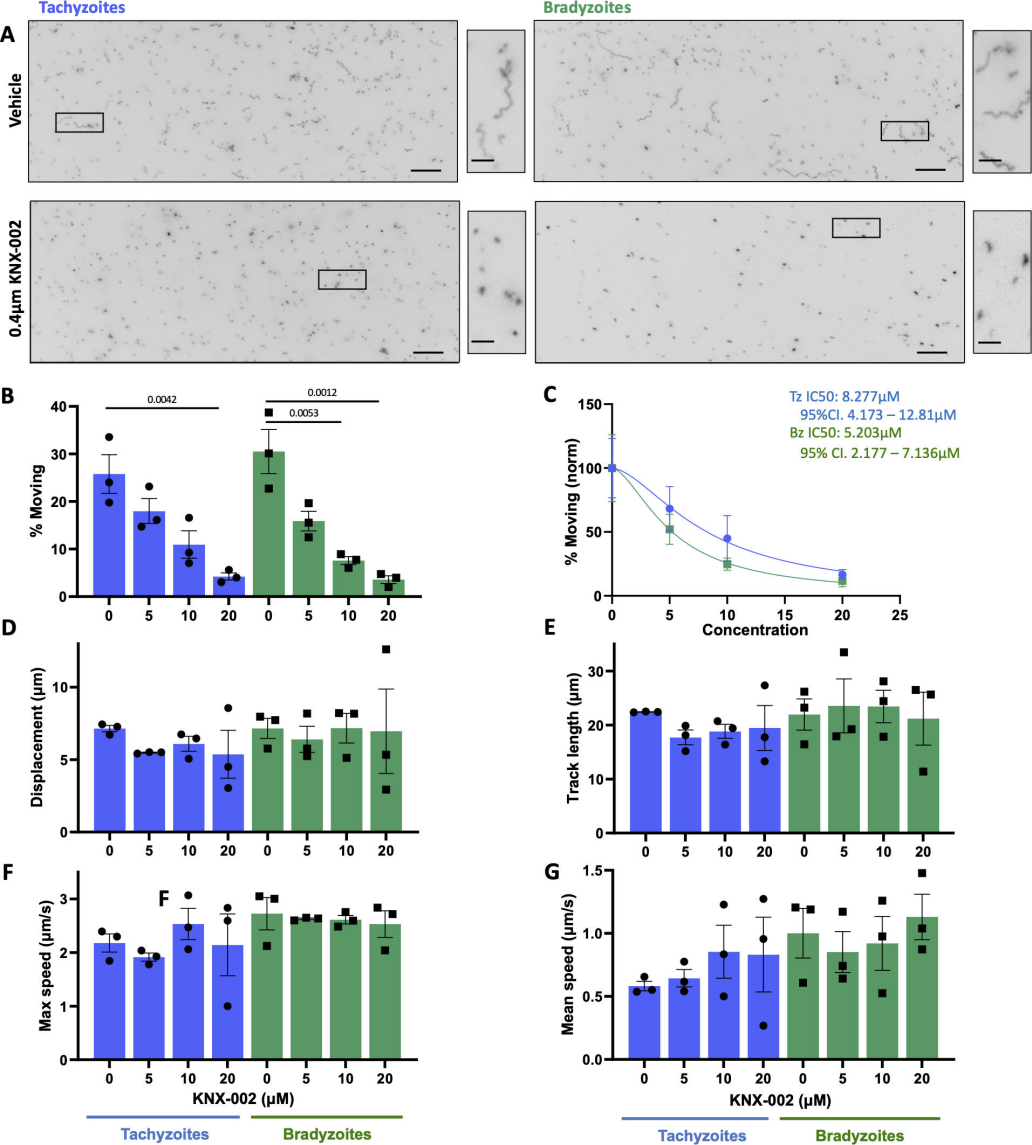
Comparison of tachyzoite and bradyzoite motility in the presence of KNX-002. (**A**) Representative maximum intensity projections of tachyzoites and bradyzoites moving through Matrigel during 60 s of imaging in the absence (top two panels) or presence (bottom two panels) of 20 µM KNX-002; scale bar = 40 µm. Insets (black boxes) are magnified, rotated, and displayed to the right of each full field of view; scale bar = 10 µm. (**B**) Percentage of tachyzoites moving >2.5 µm and bradyzoites moving >2.8 µm during 60 s of imaging. (**C**) The IC50 values for the data shown in panel B were calculated for tachyzoites (blue) and bradyzoites (green); % motility for each data set was normalized to 100% in the sample incubated without KNX-002. No significant difference was seen in the tachyzoite versus bradyzoite response to treatment, as indicated by the overlapping 95% confidence intervals (CIs) of the calculated IC50 values. (**D–G**) For all parasites that exceeded the 2.5/2.8 µm displacement thresholds, the following median trajectory parameters were quantified: (D) displacement, (**E**) track length, (**F**) maximum speed, and (G) mean speed. On the graphs, each data point represents a biological replicate consisting of two to three technical replicates. Bar height shows the mean, and error bars show the s.e.m. of the biological replicates. The responses of tachyzoites and bradyzoites at each concentration of KNX-002D were compared using unpaired Student’s *t*-tests; no significant differences were identified between the two stages at any dose. The numbers of parasites analyzed in panels B–G were 2,112; 1,186;1,251; and 715 (tachyzoites 0, 5, 10, and 20 µM KNX-002, respectively); and 3,108; 1,199; 868; and 600 (bradyzoites 0, 5, 10, and 20 µM KNX-002, respectively). The responses of tachyzoites or bradyzoites to treatment with each concentration of compound were compared to the vehicle control (0) with an ordinary one-way analysis of variance and Tukey’s correction for multiple comparisons; only statistically significant differences (*P* < 0.05) are shown.

We next wanted to determine if known modulators of tachyzoite motility have comparable effects on bradyzoite motility. CytoD inhibits actin polymerization and has a well-documented inhibitory effect on tachyzoite motility in 2D (49, 50). KNX-002 is a recently described inhibitor of the myosin motor TgMyoA that also inhibits tachyzoite motility (30). Given the central role of actin and TgMyoA in the motility of multiple apicomplexan zoites (25), we predicted that bradyzoite motility would also be inhibited by these compounds. This was indeed the case: in all measured parameters of motility and across all concentrations of compound, tachyzoites and bradyzoites were similarly inhibited by both CytoD and KNX-002 (Fig. 4 and 5, respectively). Quantification of the effect of the compounds on the proportion of parasites moving yielded consistent IC50 values of 0.12 and 0.10 µM CytoD and 8.28 and 5.20 µM KNX-002 for tachyzoites and bradyzoites, respectively, in each case with overlapping confidence intervals (Fig. 4C and 5C).

#### Tachyplegin

**Fig 6:**
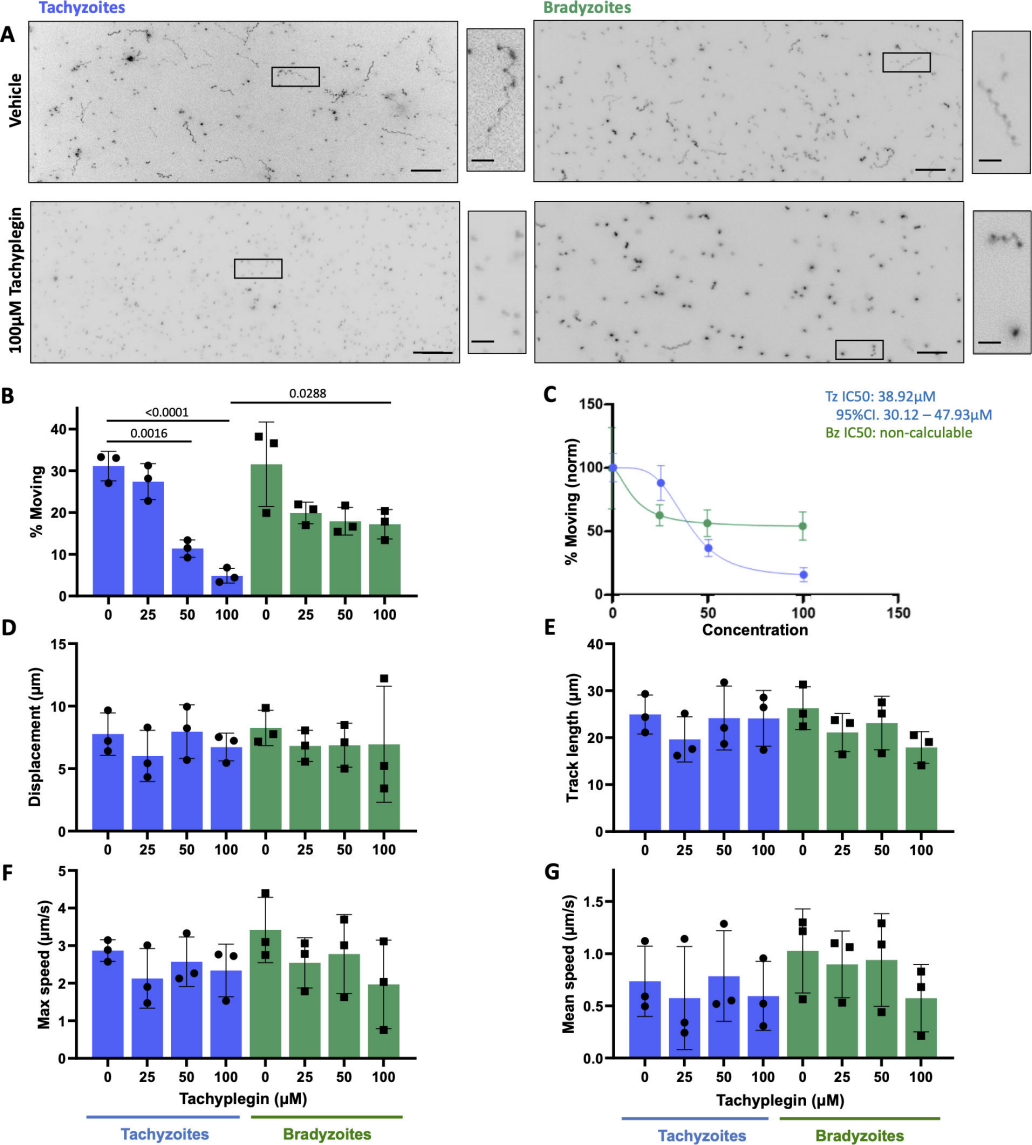
Comparison of tachyzoite and bradyzoite motility in the presence of tachyplegin. Representative maximum intensity projections of tachyzoites and bradyzoites moving through Matrigel during 60 s of imaging in the absence (top two panels) or presence (bottom two panels) of 100 µM tachyplegin; scale bar = 40 µm. Insets (black boxes) are magnified, rotated, and displayed to the right of each full field of view; scale bar = 10 µm. (**B**) Percentage of tachyzoites moving >2.5 µm and bradyzoites moving >2.8 µm during 60 s of imaging. (**C**) The IC50 value for the % motility data shown in panel B was calculated for tachyzoites (blue) to be 38.92 µM; inhibition of bradyzoite motility (green) was insufficient to calculate an IC50. The % motility for each data set was normalized to 100% in the sample incubated with no Tachyplegin. (**D–G**) For all parasites that exceeded the 2.5/2.8 µm displacement thresholds, the following median trajectory parameters were quantified: (D) displacement, (**E**) track length, (**F**) maximum speed, and (G) mean speed. On the graphs, each data point represents a biological replicate consisting of two to three technical replicates. Top of the bars show the mean, and error bars show the s.e.m. of the biological replicates. The numbers of parasites analyzed in panels B–G were 2,043; 1,552; 891; and 755 (tachyzoites 0, 25, 50, and 100 µM tachyplegin, respectively); and 1,265; 883; 1,632; and 1,474 (bradyzoites 0, 25, 50, and 100 µM tachyplegin, respectively). The responses of tachyzoites and bradyzoites at each concentration of tachyplegin were compared using unpaired Student’s *t*-tests; a significant difference was observed only when motilities of the two stages were compared for parasites treated with 100 µM tachyplegin. The responses of tachyzoites or bradyzoites to treatment with each concentration of compound were compared to the vehicle control (0) with an ordinary one-way analysis of variance and Tukey’s correction for multiple comparisons; only statistically significant differences (*P* < 0.05) are shown.

Tachyplegin is an inhibitor of tachyzoite motility that targets myosin light chain-1 (MLC1), another critical component of the parasite’s myosin motor complex (22, 51, 52). Tachyplegin causes a steady dose-dependent decrease in the proportion of tachyzoites that move in 3D, over the range of 0–100 µM compound (51) (Fig. 6A through C, tachyzoites). In contrast, while 25 µM tachyplegin causes an initial decrease in the proportion of bradyzoites moving, increasing amounts of compound beyond 25 µM do not inhibit motility further (Fig. 6A through C, bradyzoites). As previously reported for RH strain tachyzoites (51), we saw no significant difference in any of the other calculated median motility parameters in response to up to 100 µM tachyplegin, in either tachyzoites or bradyzoites of the PRU strain (Fig. 6D through G). For tachyzoites, treatment with tachyplegin also had no impact on the distributions of these motility parameters (Fig. S3). However, while bradyzoites treated with 100 µM tachyplegin could still move, they were less able to move for long displacement distances than vehicle-treated parasites, as evident from the markedly truncated violin plot and a statistically significant difference in the displacement length for parasites in the 95th percentile within each population (Fig. S3A). Thus, tachyplegin does not ablate bradyzoite motility to the same extent as tachyzoite motility at this higher dose but does reduce the distance the parasites can move (displacement) compared to untreated bradyzoites.

#### Enhancer 5

Enhancer 5/compound 130038 was previously shown to increase tachyzoite motility in 2D by stimulating intracellular calcium release, which leads to increased microneme secretion and TgMyoA phosphorylation (53, 54). Enhancer 5 (100 µM) increased the proportion of tachyzoites moving in the 60 s 3D motility assay, from 24% ± 3% to 52% ± 4%, but had no statistically significant effect on the proportion of bradyzoites moving (Fig. S4A and B). The effect of the enhancer is apparently specific to the proportion of tachyzoites moving: all other measured parameters of both tachyzoite and bradyzoite motility were unaffected by treatment with 100 µM enhancer 5 (Fig. S4C through F).

#### Calcium ionophores

**Fig 7:**
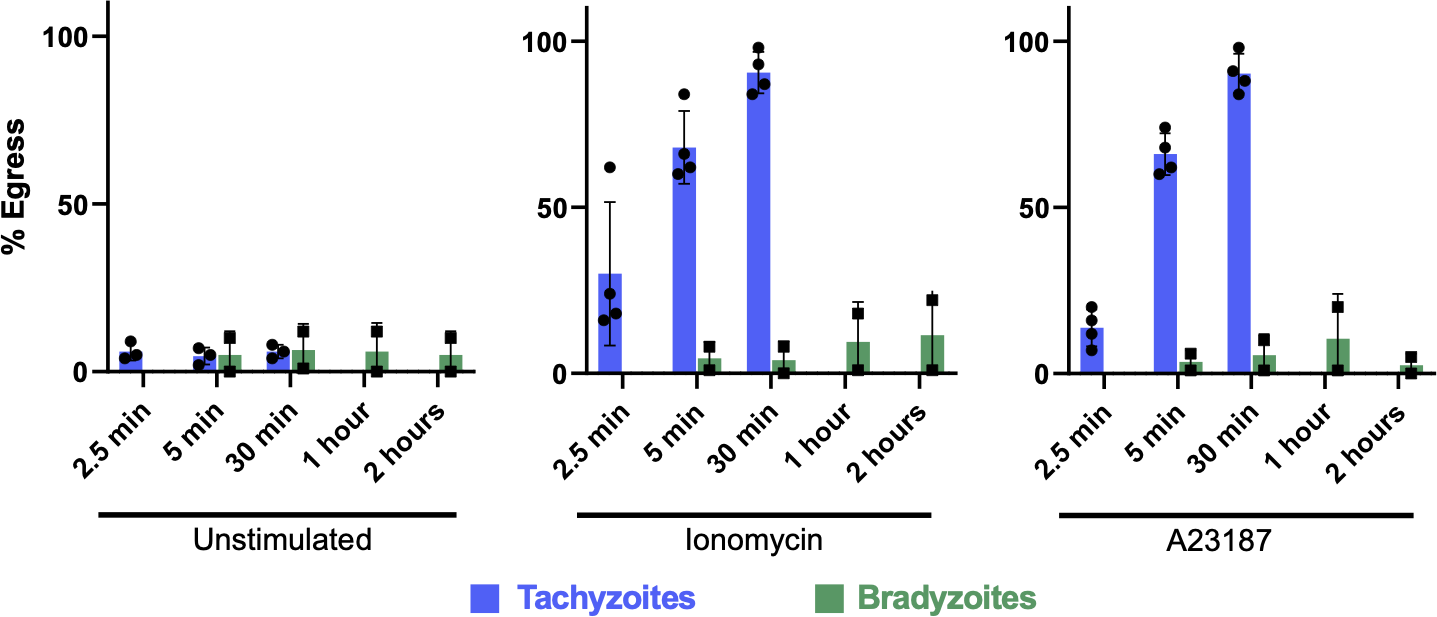
Bradyzoite egress is not calcium stimulated. Following treatment with the calcium agonists ionomycin (middle graph) and A23187 (right graph), tachyzoites (blue bars) rapidly leave the host cells in which they reside, whereas extended treatment with these agonists induces little to no bradyzoite (green bars) egress.

It has been previously reported that egress of type II (Me49) tachyzoites from host cells is triggered by stimulation with calcium agonists, but egress of Me49 bradyzoites is not (28). We confirmed this observation with PRU parasites, showing that even extended (30 min) exposure to either ionomycin or A23187 caused no detectable egress of PRU bradyzoites compared to >80% egress by tachyzoites (Fig. 7).

Taken together, these data demonstrate that while some pharmacological agents targeting the parasite’s motor machinery or the signaling mechanisms underlying motility have similar effects on tachyzoites and bradyzoites, other compounds have distinctly different effects on these two life cycle stages.

## DISCUSSION

Differentiation of *T. gondii* to bradyzoites and encystment in cells of the brain and other tissues results in a chronic infection. We show here that the bradyzoites within the cysts are primed to move upon excystation. When released from the cyst by the combination of mechanical shear and a short acid pepsin digestion, bradyzoites are as motile as tachyzoites in the 60–80 s 3D motility assay used here. Further studies would be required to assess their relative longevity and/or to determine if carrying out the motility assays under different conditions (e.g*.*, gliding on a 2D surface) yields similar results, but we have shown that with the isolation method and assay used here, bradyzoites move as efficiently as tachyzoites. Bradyzoites are also at least as invasive as tachyzoites into a variety of cell types. The higher levels of invasion of bradyzoites relative to tachyzoites into CACO-2 and HCT-8 cells may reflect an enhanced ability of bradyzoites to invade cells of the gut epithelia. Bradyzoites also show an increased ability to transmigrate across polarized epithelial cell monolayers (MDCK and HCT-8 cells). Since establishment of an infection following ingestion of bradyzoite-containing cysts requires the parasites to rapidly leave the cyst, migrate to the gut wall, and either actively invade into the gut epithelial cells or transmigrate between them to access the lamina propria, the enhanced ability of bradyzoites to invade gut cells and transmigrate across polarized epithelia may be adaptive traits in this parasite life cycle stage. Enhanced transmigration ability appears to be cell type specific, however, as neither bradyzoites nor tachyzoites transmigrate efficiently across CACO-2 cell monolayers. A limitation of these data is the use of monocultures of colonic carcinoma cells, which offer experimental consistency but differ metabolically and structurally from the complex cellular environment of the small intestine, where infection normally occurs. Further studies of stage-specific invasion into and transmigration across epithelial monolayers derived from complex mixtures of cell populations (e.g*.*, open format enteroids [55]) from different gut regions may reveal different cell types, tight junction properties, and/or areas of the gut that are particularly susceptible to bradyzoite infection, differences that homogeneous monolayers of cells fail to fully capture.

Two of the three tachyzoite motility inhibitors we tested (CytoD and KNX-002) show similar activity against bradyzoites and tachyzoites, suggesting conservation of an actin-myosin-based motility machinery between the two stages. Why then might tachyplegin, which targets a key component of this machinery—MLC1 (51)—have a different effect on tachyzoites and bradyzoites? It is possible that the uptake, metabolism, or extrusion of tachyplegin is different in the two life stages. An alternative hypothesis of more mechanistic interest is that the differential activity is due to differences in the phosphorylation of MLC1 in tachyzoites and bradyzoites. TgMLC1 is phosphorylated on at least nine distinct sites in tachyzoites (56). Intriguingly, two of these phosphorylation sites (S55 and S57) are located in close proximity to the tachyplegin binding site in the protein (C58) (51) and could conceivably affect compound binding. Furthermore, calcium-stimulated egress of tachyzoites is accompanied by the rapid phosphorylation of more than 50 proteins, including S55 of MLC1 (57). Perhaps the different signaling pathways that underlie tachyzoite egress and bradyzoite excystation (28) result in a different MLC1 phosphorylation profile and therefore a different sensitivity to tachyplegin. Further work will be required to test this specific hypothesis and to determine whether other known inhibitors of tachyzoite motility and motility-dependent processes such as invasion (58, 59) also show differential effects on tachyzoites and bradyzoites.

The work reported here confirms and extends previous work showing that the signaling underlying host cell egress by tachzyoites is different from the signaling underlying egress of bradyzoites from cysts. Not only are bradyzoites less responsive to calcium signaling within the cyst, but once released they are also less sensitive to the effects of enhancer 5. Enhancer 5 increases tachyzoite motility through an effect on calcium signaling and microneme secretion (54) but does not significantly increase bradyzoite motility in our 3D assay. This indicates that not only are encysted bradyzoites less responsive to calcium stimuli but that recently excysted bradyzoites show dampened responses to calcium stimuli as well.

Taken together, the data presented here demonstrate that although the motility and motility-associated behaviors of tachyzoites and bradyzoites are similar in many respects, they also differ in ways that may be adaptive for that particular life cycle stage. Tachyzoites and bradyzoites also show differing sensitivity to compounds targeting the parasite’s motor machinery and the signaling processes underlying its activation, and this needs to be considered in drug development efforts (29–33) targeting *T. gondii* motility and motility-dependent processes.

## SUPPLEMENTAL MATERIAL

Figure S1 msphere.00855-24-s0001.tif Comparison of RH and PRU tachyzoite motility.

Figure S2 msphere.00855-24-s0002.tif Comparison of the motility parameter distributions for *in vitro*- versus *in vivo*-derived bradyzoites.

Figure S3 msphere.00855-24-s0003.tif Comparison of the motility parameter distributions for tachyzoites and bradyzoites treated with tachyplegin.

Figure S4 msphere.00855-24-s0004.tif Comparison of tachyzoite and bradyzoite motility in the presence of enhancer 5.

Supplemental figure legends msphere.00855-24-s0005.pdf Legends to Figures S1-S4.

ASM does not own the copyrights to Supplemental Material that may be linked to, or accessed through, an article. The authors have granted ASM a non-exclusive, world-wide license to publish the Supplemental Material files. Please contact the corresponding author directly for reuse.
