# Supplementary figures and images for: Motility-dependent processes in Toxoplasma gondii tachyzoites and bradyzoites: same same but different
Source: mSphere. 2025 Feb 12;10(3):e00855-24. doi: 10.1128/msphere.00855-24 (PMC11934331; doi:10.1128/msphere.00855-24)

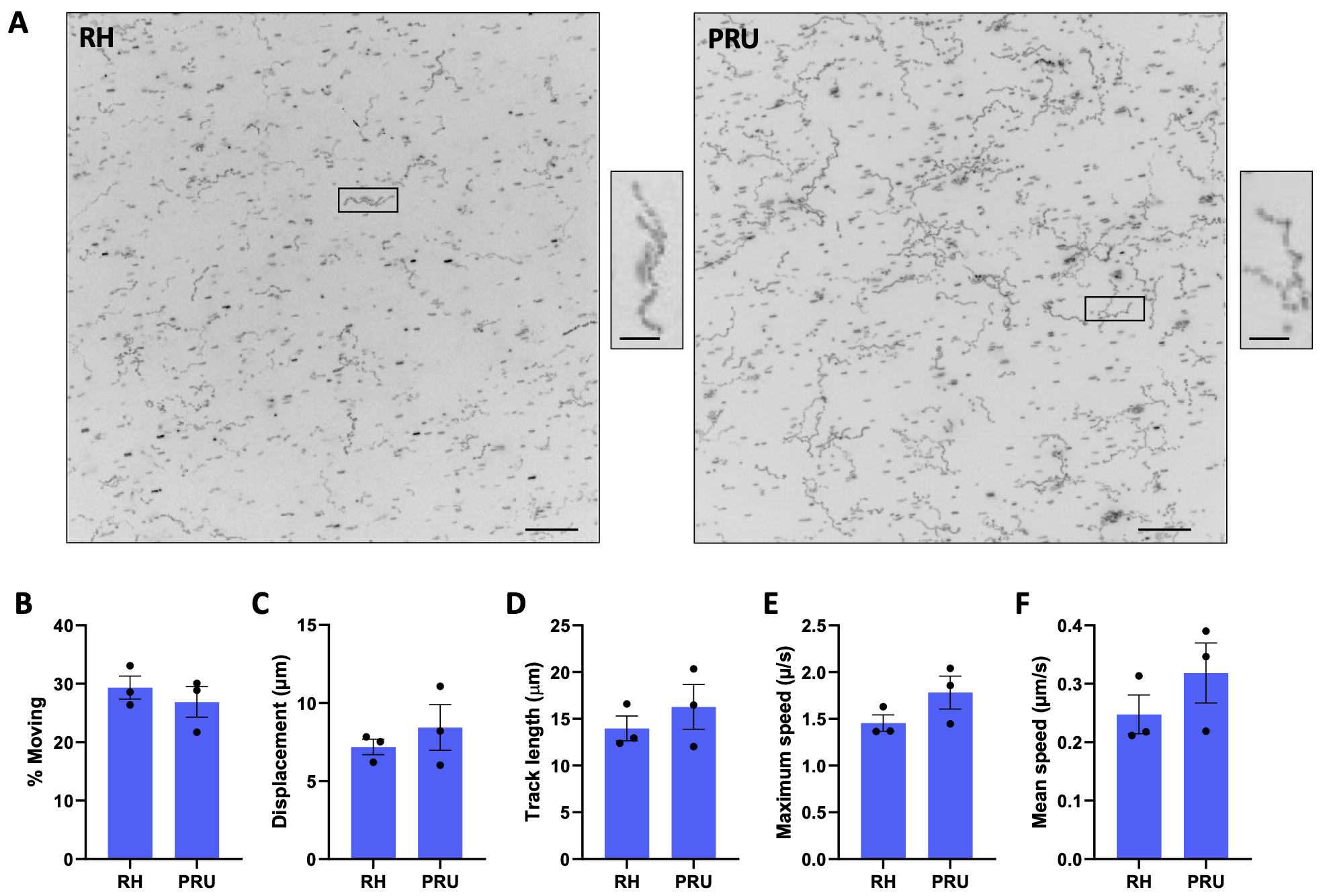

Supplement: Figure S1 — Comparison of RH and PRU tachyzoite motility. [file msphere.00855-24-s0001.tif]

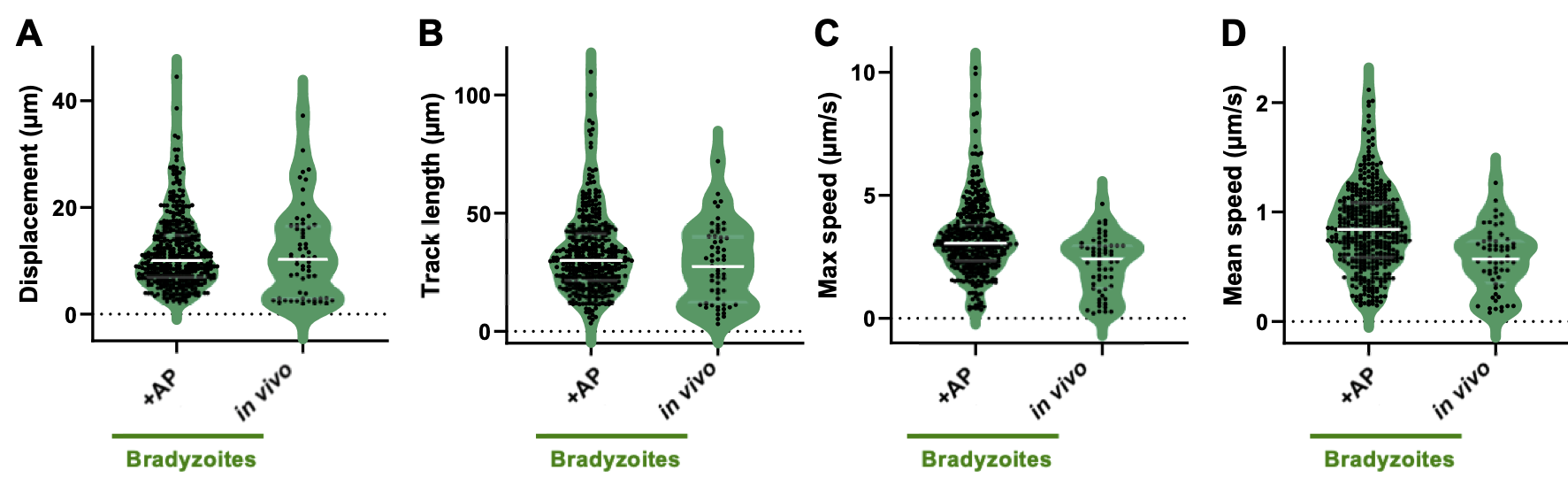

Supplement: Figure S2 — Comparison of the motility parameter distributions for in vitro- versus in vivo-derived bradyzoites. [file msphere.00855-24-s0002.tif]

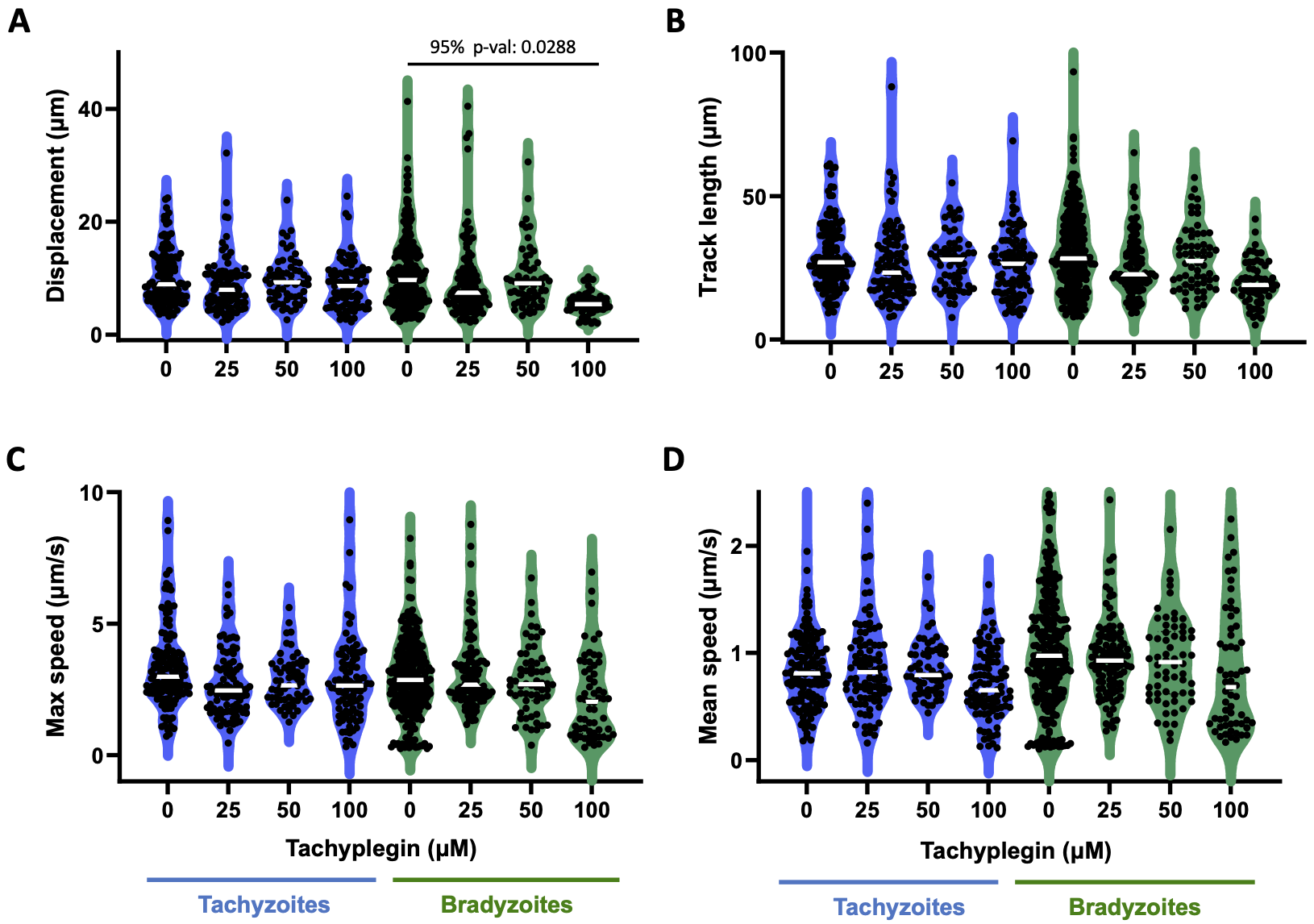

Supplement: Figure S3 — Comparison of the motility parameter distributions for tachyzoites and bradyzoites treated with tachyplegin. [file msphere.00855-24-s0003.tif]

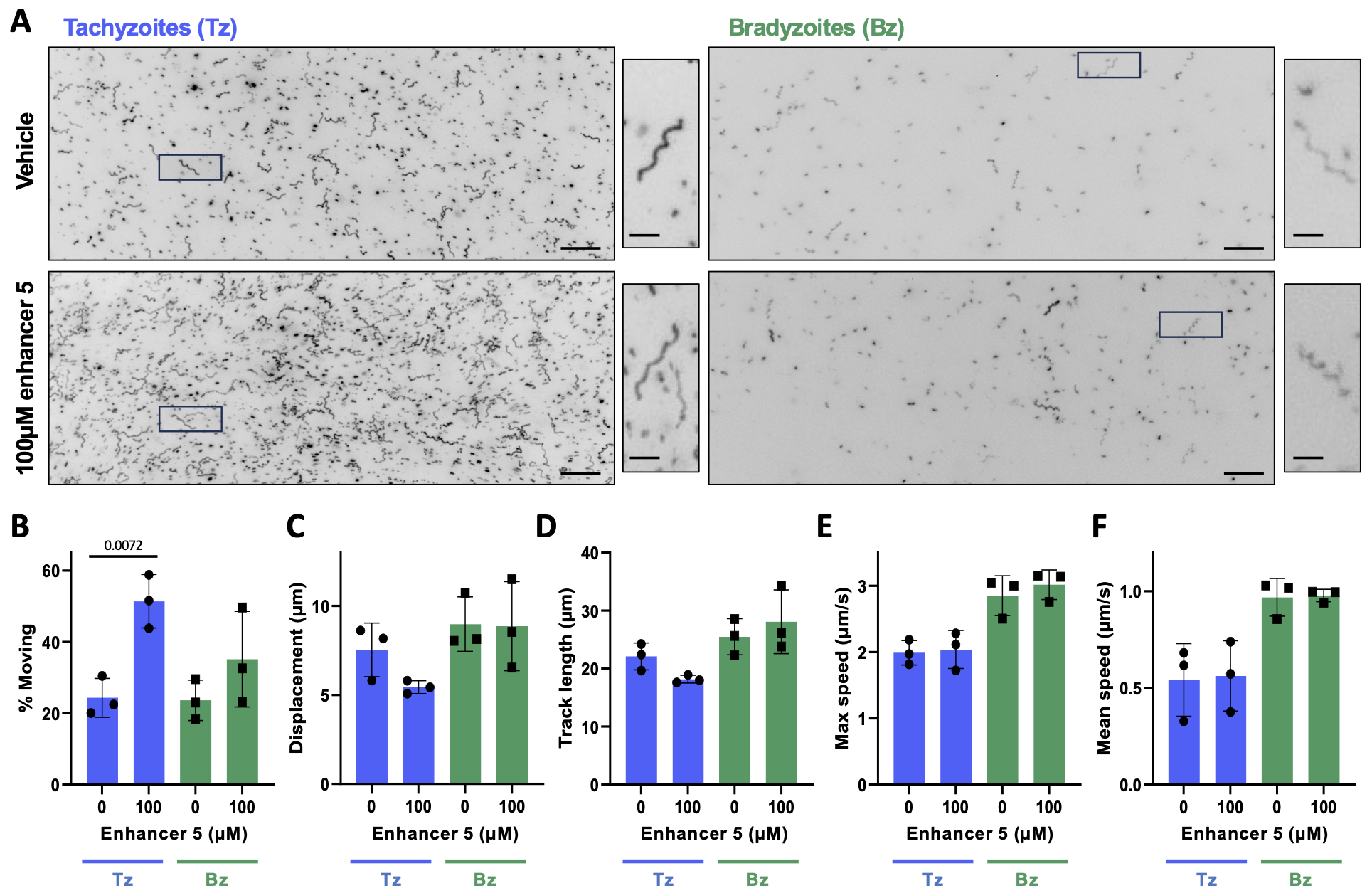

Supplement: Figure S4 — Comparison of tachyzoite and bradyzoite motility in the presence of enhancer 5. [file msphere.00855-24-s0004.tif]
