# Supplementary material for: Motility-dependent processes in Toxoplasma gondii tachyzoites and bradyzoites: same same but different
Source: mSphere. 2025 Feb 12;10(3):e00855-24. doi: 10.1128/msphere.00855-24 (PMC11934331; doi:10.1128/msphere.00855-24)
Supplement: Supplemental figure legends — Legends to Figures S1-S4. [file msphere.00855-24-s0005.pdf]

Motility-dependent processes in *Toxoplasma gondii* tachyzoites and bradyzoites: same same but different

RS Kent and GE Ward

Supplemental Figure legends

Supplementary figure 1. Comparison of RH and PRU tachyzoite motility.

(A) Representative maximum intensity projections of RH (left panel) and PRU (right panel) tachyzoites moving through Matrigel during 60 seconds of imaging; scale bar = 40μm. Insets (black boxes) are magnified, rotated and displayed to the right of each full field of view; scale bar 10μm. (B) Percentage of parasites moving > 2.5 μm during 60 seconds of imaging; total number of parasites analyzed = 9064 (RH) and 7788 (PRU). (C-F) For all moving parasites, the following median trajectory parameters were quantified: C) displacement (distance from first to last point); D) track length; E) maximum speed achieved across the entire track; and F) mean speed. Each pair of motility parameters (RH vs PRU) was compared using an unpaired students t-test, and no significant difference in these parameters was identified between the strains. On the graphs, each data point represents one of three biological replicates, each consisting of 2 – 4 technical replicates. Bar height shows the mean and error bars show the s.e.m. of the biological replicates.

Supplementary figure 2. Comparison of the motility parameter distributions for *in vitro- vs. in vivo*-derived bradyzoites.

(A) Displacement, (B) Track length, (C) Maximum speed and (D) Mean speed, for all motile parasites in the *in vitro* (plus acid pepsin digest) and *in vivo* populations, plotted as violin plots. The median of each distribution is shown as a horizontal white line. Using unpaired students t-tests, no significant differences were identified between the two populations for any of the four parameters when comparing the median, 5% or 95% values.

Supplementary figure 3. Comparison of the motility parameter distributions for tachyzoites and bradyzoites treated with tachyplegin.

Tachyzoites and bradyzoites were treated with increasing doses of tachyplegin. The motility parameters compared were (A) displacement, (B) track length (C) maximum speed achieved and (D) mean speed. The 5th and 95th percentile values for each stage and compound concentration were compared to the vehicle control (0) with an ordinary one-way ANOVA and Tukeys correction

for multiple comparisons. The only statistically significant difference was a decrease in the 95th percentile of bradyzoite displacement, comparing 100µM tachyplegin to vehicle control.

Supplementary figure 4. Comparison of tachyzoite and bradyzoite motility in the presence of enhancer 5.

(A) Representative maximum intensity projections of tachyzoites (Tz) and bradyzoites (Bz) moving through Matrigel during 60 seconds of imaging in the presence of 100µM enhancer 5; scale bar = 40µm. Insets (black boxes) are magnified, rotated and displayed to the right of each full field of view; scale bar 10µm. (B) Percentage of tachyzoites moving > 2.5µm and bradyzoites moving > 2.8µm during 60 seconds of imaging. (C-F) For all parasites that exceeded the 2.5/2.8 µm displacement thresholds, the following median trajectory parameters were quantified: C) displacement; D) track length; E) maximum speed; and F) the mean speed. Each data point represents a biological replicate consisting of 2-4 technical replicates; top of the bars show the mean and error bars show the s.e.m. of the biological replicates. The number of parasites analyzed in B-F was 2359, 2810 (Tachyzoites 0, 100µM enhancer 5 respectively) and 1743, 2109 (Bradyzoites 0,100µM enhancer 5 respectively). The response of tachyzoites and bradyzoites to enhancer 5 was compared to vehicle only (0) using unpaired students t-tests; only significant differences ($p<0.05$) are shown.
